# Bacterial endophytic community composition varies by hemp cultivar in commercially sourced seed

**DOI:** 10.1111/1758-2229.13259

**Published:** 2024-04-22

**Authors:** Jack Davies, Sarah Hawkins, Ana Winters, Kerrie Farrar

**Affiliations:** ^1^ Institute of Biological, Environmental & Rural Sciences (IBERS) Aberystwyth University Aberystwyth UK

## Abstract

The seed‐endophytic bacterial community is a potentially beneficial and heritable fraction of the plant microbiome. Its utilization as a sustainable crop improvement strategy could be especially valuable for species such as hemp, where production is being scaled up and new challenges will be faced in managing crop productivity and health. However, little is known about the makeup and variation of the hemp seed microbiome. This study profiled the endophytic bacterial communities harboured by 16 hemp cultivars sourced from commercial suppliers in Europe. A 16S rDNA amplicon sequencing approach identified 917 amplicon sequence variants across samples. Taxonomic classification of sequences revealed 4 phyla and 87 genera to be represented in the dataset. Several genera were widespread while some were specific to one or a few cultivars. *Flavobacterium*, *Pseudomonas*, and *Pantoea* were notable in their high overall abundance and prevalence, but community composition was variable and no one taxon was universally abundant, suggesting a high degree of flexibility in community assembly. Taxonomic composition and alpha diversity differed among cultivars, though further work is required to understand the relative influence of hemp genetic factors on community structure. The taxonomic profiles presented here can be used to inform further work investigating the functional characteristics and potential plant‐growth‐promoting traits of seed‐borne bacteria in hemp.

## Graphical abstract

We present the first taxonomic characterization of the hemp (*Cannabis sativa*) bacterial seed microbiome using 16S rDNA amplicon sequencing. Seed‐endophytic community composition and diversity varied among 16 commercially available hemp cultivars, though some bacterial taxa were highly prevalent across samples.

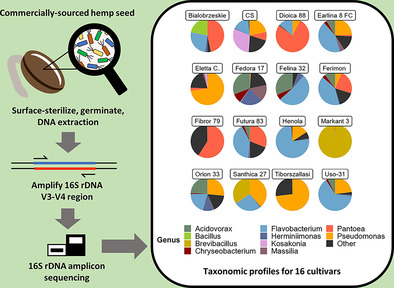

## INTRODUCTION

Industrial hemp (*Cannabis sativa*), distinguished from medical cannabis by its low tetrahydrocannabinol content, is a multiuse crop of renewed interest in research, industry, and agriculture. Many attractive properties of its stem biomass and seeds make hemp suited to numerous end uses. Its versatility, combined with its high sustainability as a high‐yielding, low‐input crop (Tang et al., 2016), has driven a resurgence in hemp cultivation (Crini et al., 2020) and hemp tonnage and acreage has been increasing worldwide (Schluttenhofer & Yuan, 2017). Hemp has been a source of fibres for thousands of years (Small, 2015) and production continues to this day, with numerous end uses including textiles, fabrics, paper, insulation, and building materials (Crini et al., 2020; Rehman et al., 2021). The high biomass of hemp also makes it suitable for bioenergy production (Finnan & Styles, 2013) and it has been considered as a source of bioethanol (Zhao et al., 2020), biogas (Kreuger et al., 2011) and solid biofuel for combustion (Prade et al., 2011). In addition to the biomass component, hemp seed has many uses in the human food, cosmetics, and animal feed industries (Crini et al., 2020). Hempseed oil has high nutritional value, containing protein, dietary fibre, vitamins, minerals, all essential amino acids, and an optimal ratio of omega‐3 and omega‐6 acids (Frassinetti et al., 2018; Xu et al., 2021). Consumption of hempseed is associated with improved cardiovascular health (Rodriguez‐Leyva & Pierce, 2010). While hemp has great potential, many challenges will be faced as production is scaled up and cultivation spreads into new areas. Hemp cultivars may need to be optimized for specific environments or end uses.

Microbiome engineering, with the aim of exploiting plant‐associated microbes to enhance yield, quality, and resilience, represents a sustainable, complementary strategy to traditional breeding approaches. Plant‐growth‐promoting rhizobacteria have been found to enhance hemp growth (Islam et al., 2023; Lyu et al., 2022; Pagnani et al., 2018). The hemp microbiome also has unique roles in mediating the retting process (Liu et al., 2017; Ribeiro et al., 2015; Zhang et al., 2008), crucial for natural fibre production and which determines fibre quality and processing efficiency (Law et al., 2020). Endophytic bacteria are known in other plants to increase host tolerance of sub‐optimal environmental conditions, increase resistance to disease, and improve nutrient acquisition (Gomes Bomfim et al., 2020; Mukherjee et al., 2020; Rahman et al., 2018; Shahzad et al., 2017).

While some seed‐transmitted bacteria can have deleterious effects on their host (Barret et al., 2016), many bacterial endophytes inhabiting the seed have been found to improve plant growth and health (Truyens et al., 2015). The plant‐beneficial traits of these seed‐endophytic bacteria, along with their potential for vertical transmission (Johnston‐Monje et al., 2021), have attracted increasing interest for crop improvement. Seed‐transmitted microbes are uniquely placed to promote early plant development, including germination and seedling growth (Goggin et al., 2015; Verma et al., 2017; White et al., 2018). Seed endophytes can contribute heavily to the microbiomes of young plants (Moroenyane et al., 2021) up to at least 2 months after germination (Johnston‐Monje et al., 2021). Indications of seed‐to‐seed transmission of endophytes (Johnston‐Monje & Raizada, 2011; Rodríguez et al., 2020; Walitang et al., 2019) suggest some seed endophytes persist throughout the plant's lifecycle, potentially conferring long‐term benefits. Seed‐derived bacterial inoculants can improve plant growth by diverse mechanisms including phytohormone and ACC deaminase synthesis, phosphorous solubilization, and enhanced acquisition of other nutrients, as well as antagonistic effects against fungal pathogens (Johnston‐Monje & Raizada, 2011; Li et al., 2020; Mukherjee et al., 2020; Rahman et al., 2018; Shahzad et al., 2017; Verma et al., 2017; White et al., 2018; Xu et al., 2014).

The hemp seed microbiome represents a promising target from improvement owing to the potentially lifelong and heritable benefits of seed endophytes. In addition, microbial activity may also modulate plant production of secondary metabolites (Taghinasab & Jabaji, 2020), with potential consequences for hemp seed use in food and cosmetic properties. Improving our understanding of the hemp seed microbiome could enable targeted microbiome engineering to improve hemp growth and end use properties. Culturable bacteria have previously been isolated from hemp seed (Dumigan & Deyholos, 2022; Gabriele et al., 2022; Scott et al., 2018). However, such methods may not capture the full bacterial diversity as a large proportion may not be amenable to culturing techniques. In addition, the level of variation in seed microbiome composition among hemp cultivars is unknown. Seed microbiome assembly is expected to depend on multiple factors at different scales, including filtering by the environment and host, as well as species interactions and stochastic factors (Bergmann & Leveau, 2022).

To undertake a more comprehensive survey of the hemp seed microbiome, this study applied a culture‐independent amplicon sequencing method to profile the bacterial communities harboured by the seeds of 16 hemp cultivars sourced from six European suppliers. The aims of the study were to explore microbial diversity among hemp seed accessions and analyse variation in community composition. By including a surface‐sterilization step, this study excludes epiphytes and focuses exclusively on endophytic bacteria that inhabit the internal tissues of the seed.

## EXPERIMENTAL PROCEDURES

### Seed material

Seed from the cultivars ‘Carmagnola Selezionata’ (henceforth CS), ‘Elleta Campana’ and ‘Tiborszallasi’ were obtained from the Council for Agricultural Research and Economics (Italy); ‘Dioica 88’, ‘Earlina 8 FC’, ‘Fedora 17’, ‘Felina 32’, ‘Ferimon’, ‘Fibror 79’, ‘Futura 83’, ‘Orion 33’ and ‘Uso‐31’ from HEMPit (France); ‘Santhica 27’ from Hempoint (Czechia); ‘Markant 3’ from Vandinter Semo (The Netherlands); and ‘Bialobrzeskie’ and ‘Henola’ seed were sampled from a seed testing station in Poland. Seed from HEMPit and Hempoint was produced in France. The country of production was unknown for material sourced from CREA and Vandinter Semo. Four cultivars were dioecious and the others were monoecious (Table S1). The cultivars ‘Futura 75’ and ‘Santhica 70’ were initially included but were filtered out of the dataset because of their much lower read count across all replicates, relative to other cultivars. Details of the commercial breeding programmes and the genetic relatedness of cultivars were not available.

### Sample preparation

Seeds were surface sterilized without dehusking. Seeds were transferred to commercial bleach diluted to 10% in sterile distilled water (SDW). After 15 min, seeds were removed from the bleach and rinsed in SDW six to eight times. The rinsing was done by placing seeds in a 15 mL tube containing SDW, shaking the seeds intermittently, then decanting out the water and pouring in fresh SDW. Seeds were left to soak in SDW overnight, then transferred into a fresh 10% bleach solution and shaken intermittently for 30 min, and finally rinsed in SDW as previously. A sample of water from the final rinse was retained and its sterility was verified by polymerase chain reaction (PCR; see Supplementary material S1 for protocol).

Three biological replicates, each consisting of 3 seeds, were prepared for each of the 16 cultivars. Sterilized seeds were transferred onto moist, sterilized filter paper and sealed within plates, and left to germinate in a dark space for 10 days, by which point germination had clearly started. Germinating seeds before DNA extraction can increase the bacterial diversity captured in amplicon sequencing (Thomas & Sahu, 2021). Seedlings were flash frozen in liquid nitrogen and ground with an ethanol‐sterilized mortar and pestle. Liquid nitrogen was used to keep the seedling material and equipment cold throughout. DNA extraction was performed using the FastDNA™ SPIN Kit for Soil (MP Biomedicals™, USA), following the manufacturer's protocol. DNA samples were quantified using an Invitrogen™ Qubit® 2.0 fluorometer, using the manufacturer's protocol. Samples were diluted with a goal concentration of 10 ng/μL. Residual material was destroyed to comply with licensing requirements.

### Amplification and sequencing

A detailed description of protocols for library preparation and sequencing is included within the Supplementary material S1. Briefly, PCR was conducted to amplify the V3‐V4 16S rDNA region and PCR clean‐up was performed using AMPure XP beads (Beckman Coulter™, USA). Subsequent stages of the pipeline were performed by the Earlham Institute (Norwich, UK), including index PCR, library preparation, and paired‐end sequencing on the Novaseq 6000 platform (Illumina Inc.). Fifty libraries were sequenced in total, including 48 hemp samples, and 2 negative controls exposed to DNA extraction and all subsequent steps to check for contamination.

### Data analysis

Primers were removed from reads using Cutadapt v2.6 (Martin, 2011), with untrimmed reads filtered out. Subsequent work was performed in R v4.1.2 (R Core Team, 2021). Subsequent pre‐processing was performed with the DADA2 v1.24.0 package (Callahan, McMurdie, et al., 2016) following (Callahan, Sankaran, et al., 2016). Forward and reverse reads were trimmed to lengths of 233 and 229, respectively. Reads with more than two expected erroneous base calls were filtered out. The units of amplicon analysis were amplicon sequence variants (ASVs; Callahan et al., 2017), which were inferred using the dada() function utilizing error rates were learned from the data. Forwards and reverse reads were merged. Predicted chimeric sequences were filtered out. Taxonomy was assigned to remaining sequence variants using DADA2's assignTaxonomy() function, with the SILVA rRNA database v138.1 (Quast et al., 2013) as a reference database. Species was assigned only for exact matches with the SILVA database (Edgar, 2018).

The final ASV table was imported as a phyloseq (v1.40.0) object for subsequent analysis (McMurdie & Holmes, 2013). Sequence variants assigned as chloroplast at the order level or mitochondria at the family level were removed. Probable contaminants were identified using the decontam package v1.16.0 (Callahan, McMurdie, et al., 2016), based on the prevalence of ASVs in the negative control samples. ASVs present in less than three samples were also filtered out of the dataset. Data were visualized using the ggplot2 v3.4.2 (Wickham, 2016), phyloseq, microbiome v1.18.0 (Lahti & Shetty, 2012), and microViz v0.9.1 (Barnett et al., 2021) packages. ASV richness was estimated using the rarefy() function from vegan v2.6.2 (Oksanen et al., 2022) with rarefaction to a sample of 21,667, the size of the smallest library. Shannon's diversity index and Faith's Phylogenetic Diversity (PD) index were calculated for each of 1000 ASV tables produced by repeated, random subsampling to 21,667 reads using vegan's rrarefy(), with the mean index values across iterations used as the final value for each sample. For ASV richness, a one‐way analysis of variance (ANOVA) was used to test for differences in among cultivars and pairwise comparisons were performed with Tukey's test using the agricolae package v1.3.7 (de Mendiburu, 2023). For measures of Shannon's index and Faith's PD, the residuals were found not to be normally distributed, therefore the Kruskal–Wallis test was used to test for differences in among cultivars and pairwise comparisons were performed with Dunn's tests implemented with the FSA v0.9.4 package (Ogle et al., 2023) with Benjamini–Hochberg adjustment of *p*‐values. Unrarefied data were converted to proportions before performing principal coordinate analysis (PCoA) of Bray–Curtis and weighted UniFrac distances.

Raw data of 16S rRNA gene amplicon sequences supporting the findings of this study are available in the Sequence Read Archive of NCBI under BioProject accession PRJNA1008886. The script for the analysis can be found at https://github.com/JackAlunDavies/Hemp_seed_microbiome_paper.

## RESULTS

### A small subset of taxa dominate hemp seed microbiomes

Thirty‐four ASVs were identified as contaminants by decontam based on its ‘prevalence’ method, while 66 ASVs were present in at least one negative control but they were not classified as contaminants by decontam (see Table S2). Many reads were assigned as mitochondrial (9.6%) and chloroplast (59.6%) DNA. These were filtered from the dataset along with reads assigned as contaminants, reads unclassified at phylum level and ASVs present in fewer than three samples.In total, 10,013,178 reads remained, representing 5 phyla, 5 classes, 15 orders, 28 families, 87 genera, and 917 ASVs. The median read count was 210,715 per sample.

Proteobacteria was the dominant phylum by mean relative abundance (60.6%), with Bacteroidota (27.9%), Firmicutes (11.1%), Actinobacteriota (0.5%), and Cyanobacteria (0.03%) also present. The results of a BLAST® search (Altschul et al., 1990) suggested that the single sequence variant assigned to Cyanobacteria was most likely derived from bacteria rather than host chloroplasts. Among individual samples, Proteobacteria was often the most abundant phylum (64.6% of samples), though in some samples it was Bacteroidota (27.1%) or Firmicutes (8.3%). The identity of the most abundant phylum was consistent among all 3 replicates for 11 of the 16 cultivars (Figure S4).

**FIGURE 1:**
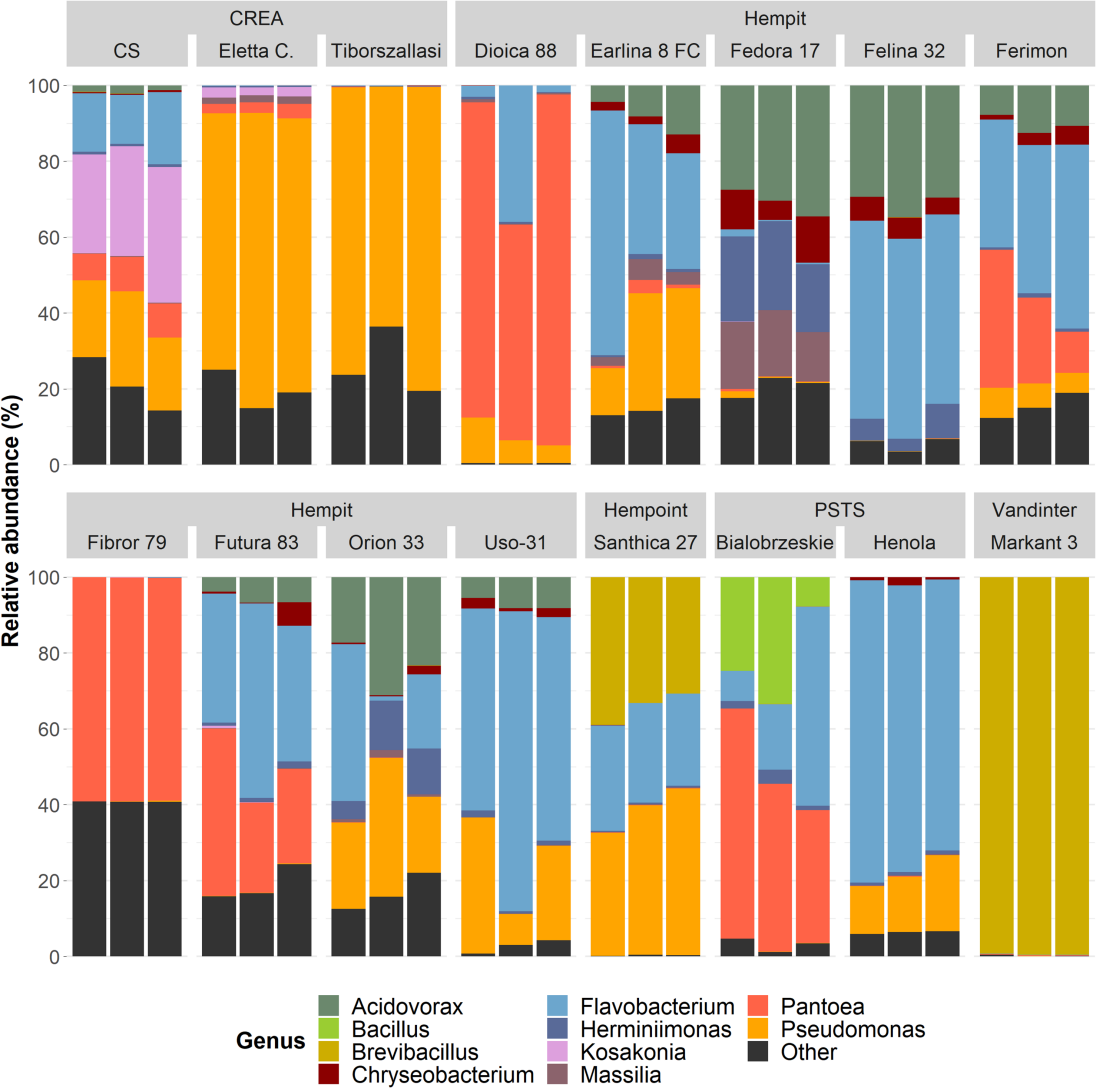
Relative abundance of bacterial genera within each sample, grouped by seed supplier and hemp cultivar. Only the top 5 most abundant genera by mean relative abundance are shown as individual colours. All other genera, as well as taxa unclassifiable to genus level, are grouped together as ‘Other’. Eletta C., Eletta Campana.

The 10 most abundant genera by mean relative abundance were (highest to lowest): *Flavobacterium* (26.1%), *Pseudomonas* (19.4%), *Pantoea* (15.7%), *Brevibacillus* (8.4%), *Acidovorax* (7.5%), *Herminiimonas* (2.9%), *Kosakonia* (2.1%), Chryseobacterium (1.8%), *Massilia* (1.5%), and *Bacillus* (1.4%). The relative abundances of these taxa are shown for each sample in Figure 1. The dominance of a relatively small number of genera was notable across the hemp seed communities. The 10 and 5 most abundant genera collectively represented 86.7% and 77.1% of reads, respectively (Figure S5). The most abundant 3 (*Flavobacterium*, *Pseudomonas*, *Pantoea*) were particularly abundant and dominated most samples, collectively representing 61.2% of total reads and >50% in 37 of 48 samples. However, the mean proportion represented by this subset varied among cultivars, from 0.3% (Markant 3) to 98.6% (Dioica 88). The dominant three genera were highly prevalent, being the only genera alongside *Herminiimonas* to have a relative abundance of ≥0.1% in ≥75% of samples. However, the relative abundance of individual genera varies substantially among samples and no genera were present in all samples. Even the most abundant genus overall, *Flavobacterium*, represented less than 1% of the community in 14 of 48 samples.

Single ASVs were mostly responsible for the high abundance of some genera. Single ASVs comprised almost all reads assigned to *Flavobacterium* (99.4% of reads), *Chryseobacterium* (99.9%), and *Herminiimonas* (96.7%). A set of ASVs contributed to the high abundance of *Pseudomonas* and *Panteoa*, which were associated with four and three ASVs, respectively, that had mean relative abundance >1%.

### Some taxa are specific to one or a few cultivars

While the dataset reveals high taxonomic diversity in the dataset at the genus level, some genera were rare. If a minimum abundance threshold of 0.1% is used to identify taxa present at a significant abundance, 24 genera do not meet this threshold in any sample. An additional 32 genera were present beyond the threshold in fewer than 10 samples, including 14 genera present in all replicates of 1 cultivar but not in any other samples: *Bacillus* (in cultivar Bialobrzeskie); *Atlantibacter*, *Lachnoclostridium*, and *Siccibacter* (CS); *Sediminihabitans*, ‘SN8’, *Stenotrophomonas*, and *Neorhizobium* (Tiborszallasi); *Variovorax* (Henola); *Cellulomonas* (Felina 32); *Raoultella* (Ferimon); *Methylobacterium‐Methylorubrum* (Earlina 8 FC); *Simplicispira* (Orion 33); and *Serratia* (Eletta Campana). Similarly, six genera (*Acinetobacter*, *Brevibacillus*, *Enterococcus*, *Kluyvera*, *Paenibacillus* and *Sanguibacter‐Flavimobilis*) were present in each replicate of two cultivars but no other sample, and two (*Allorhizobium‐Neorhizobium‐Pararhizobium‐Rhizobium* and *Erwinia*) in only three cultivars. Of these 22 genera that showed potential cultivar‐specificity, most were relatively rare where they were present (median 1.5% relative abundance) and only 2 comprised on average more than 10% of the communities where they were present: *Brevibacillus* (66.9%) and *Bacillus* (22.0%). *Brevibacillus* comprised almost the entire community in Markant 3 samples (mean 99.5% relative abundance), despite being otherwise present in only one other cultivar (Santhica 27) beyond the 0.1% abundance threshold.

### Cultivars differ in measures of alpha and beta diversity

**FIGURE 2:**
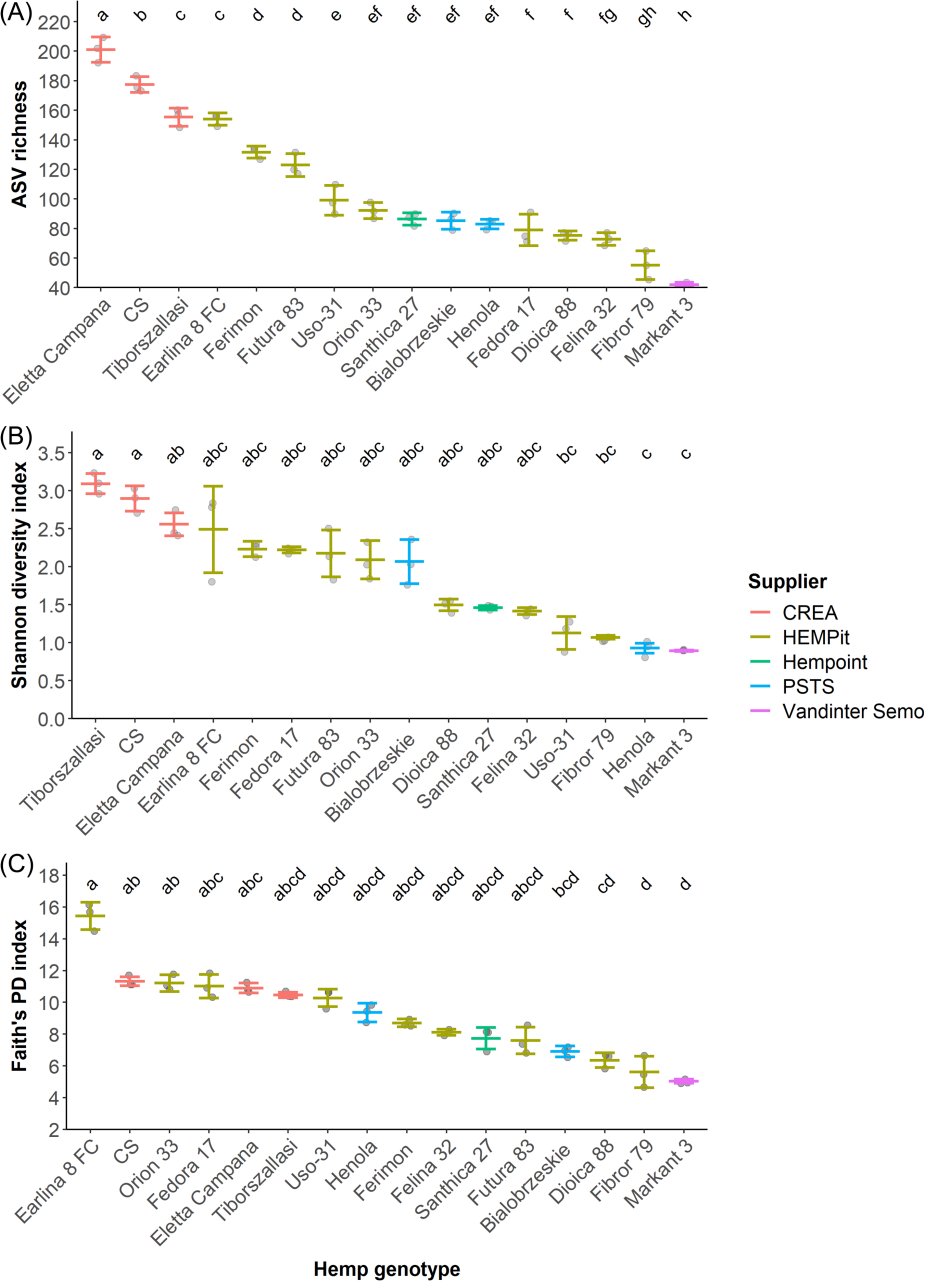
Amplicon sequence variant (ASV) richness (A), Shannon diversity index (B) and Faith's Phylogenetic Diversity (C) index by hemp cultivar. Error bars represent standard errors. Significant differences between cultivars, represented by the compact letter display, derive from the results of pairwise comparisons with *p*‐value adjustment (Benjamini–Hochberg).

ASV richness varied from 36 to 180 among samples, with a median of 88 and mean of 99.8. All three tested metrics of richness and diversity varied significantly by hemp cultivar: ASV richness (one‐way ANOVA, df = 15, *F* = 149, *p*‐value < 0.001), Shannon's diversity index (Kruskal–Wallis test, df = 15, Kruskal–Wallis chi‐squared = 43.5, *p*‐value < 0.001) and Faith's PD (Kruskal–Wallis test, df = 15, Kruskal–Wallis chi‐squared = 44.9, *p*‐value < 0.001). For each metric, pairwise comparisons showed that some cultivars differed significantly from each other (adjusted *p*‐value < 0.05; Figure 2A–C).

Dissimilarities in microbiome composition among samples were calculated as Bray‐Curtis and weighted UniFrac distances. PCoAs of the Bray–Curtis distances showed that samples from the same cultivar typically clustered more closely to each other that to samples from other cultivars; this was true for ASV‐level data (Figure S1A,B) and data agglomerated to genus level (Figure S2A,B). Similarly, samples of a given cultivar were typically seen to cluster together in the PCoA of the weighted UniFrac distances (Figure S3A,B). Overall, samples from the same cultivar generally showed a high level of consistency in the composition of their bacterial communities in terms of sequence variant diversity, taxonomic diversity, and PD.

## DISCUSSION

This study reveals the bacterial, seed‐endophytic diversity harboured across a range of European hemp cultivars. To our knowledge, this is the first study to apply a culture‐independent sequencing approach to profile the seed‐endophytic microbiome of hemp. In addition, the 16 cultivars in this study represent the largest diversity panel to which the 16S rDNA amplicon sequencing approach has been applied in any hemp microbiome study. Our results provide novel insights into the diversity and taxonomic composition of hemp seeds and the extent of variation among seed lots.

At the phylum level, our taxonomic profiles of the hemp seed microbiome are similar to those obtained in previous studies. While Cyanobacteria contributed a small number of reads, the communities were otherwise comprised exclusively of Proteobacteria, Bacteroidota, Firmicutes, and Actinobacteriota, four phyla that have been found to dominate the seed microbiomes of other plant species (Simonin et al., 2022; War et al., 2023). The five phyla in our dataset also comprise the five most abundant phyla reported across the hemp root, leaf, and flower endopheres (Wei et al., 2021). The most abundant phylum in hemp seed was Proteobacteria, which was also reported by Willman et al. (2021) to dominate hemp root, leaf and bud endospheres, alongside Bacteroidota and Actinobacteriota in the root. However, Ahmed et al. (2021) reported the hemp root and rhizosphere microbiomes to be strongly dominated by Planctobacteria (Planctomycetota), which was absent from our dataset.

The culturable bacterial community of hemp seed has been previously isolated from the cultivar Futura 75 (Gabriele et al., 2022). All stains isolated in that study were attributed to the phyla Proteobacteria, Firmicutes, and Actinobacteriota but none to Bacteroidota, the second most abundant phylum in our dataset. Of the genera included among the closest BLAST matches to the stains isolated by Gabriele et al. (2022), *Bacillus*, *Cellulomonas*, *Curtobacterium*, *Paenibacillus*, *Sphingomonas*, and *Stenotrophomonas* were also included in our dataset, but three were not: *Kocuria*, *Psychrobacillus*, and *Staphylococcus*. Our dataset included 81 genera that were not isolated by Gabriele et al. (2022), including the 3 most abundant genera in our dataset. This may reflect any combination of plant genetic and environmental effects on seed microbiome assembly (Klaedtke et al., 2016; Wassermann et al., 2022) and differences in the diversity captured by culture‐dependent and ‐independent methods (Cope‐Selby et al., 2017; Jackson et al., 2013).

Alpha diversity was found to highly variable among cultivars by each metric analysed, including the number sequence variants (ASV richness), a diversity index that incorporates the richness and evenness of ASVs (Shannon's diversity index) and a measure of phylogenetic diversity (Faith's PD). The cultivars Eletta Campana, CS, and Earlina 8 FC were among the top five by each alpha diversity metric, while Markant 3 was always the least diverse cultivar. The observed median ASV richness (90) was higher the median calculated (40) from a cross‐species meta‐analysis of similar studies profiling the microbiomes of surface‐sterilized seed (Simonin et al., 2022). It is much lower than the ASV richness figures reported from endosphere samples from the root (1141), leaf (342), and bud (181) of the hemp cultivar Tangerine (Willman et al., 2021). The seed microbiome was found to harbour lower diversity than other endophytic compartments in a culture‐based study in hemp (Scott et al., 2018). This pattern of diversity may be explained by requirements for specialist characteristics required to colonize and survive within seeds (War et al., 2023).

Eighty‐seven genera were included in the dataset, though a relatively small subset dominated most samples. The 10 most abundant genera overall collectively represented the majority of every sample (Figure 1). Furthermore, three taxa (*Flavobacterium*, *Pseudomonas*, *Pantoea*) collectively dominated most samples. *Pseudomonas* and *Pantoea* are very commonly observed in seed microbiomes—the most prevalent and abundant ASVs across 50 plant species included taxa assigned to these 2 genera (Simonin et al., 2022). The consistent recruitment of certain taxa into hemp seed microbiomes may be driven by a combination of factors such as ubiquity in the surrounding environment and possession of traits that facilitate colonization, survival, and inheritance within seed, potentially including traits that preferential selection by the host plant. *Flavobacterium*, *Pseudomonas*, and *Pantoea* each include members previously found to confer plant beneficial effects (Gontia‐Mishra et al., 2016; Walterson & Stavrinides, 2015; Weller, 2007).

Association with any one specific taxon does not seem obligate for hemp seed. No taxa are present in every sample, and even the most common taxa are absent or present at low abundance in several samples. The high flexibility of the communities is exemplified by the almost complete dominance of seed of the Markant by *Brevibacillus*, an uncommon taxon across most of the dataset. At the genus level, no fraction of the hemp seed microbiome was fully stable to be defined as a core microbiota, based on the commonly applied definition of 100% prevalence across all samples (Berg et al., 2020; Neu et al., 2021), though the high prevalence and abundance of some taxa may still suggest key ecological and functional roles within the microbiome such as those hypothesised for core taxa (Neu et al., 2021).

In all cases there was high similarity in community composition across replicates—for samples from a given cultivar, across which plant genotype and likely environmental conditions were consistent, similar assemblages of bacteria were recruited into seeds in similar proportions. This supports findings that deterministic processes are more important than stochastic factors in microbiome assembly, especially in seed (Guo et al., 2021). There was much greater variation in community composition across cultivars. These differences cannot be confidently attributed to host genotype as the cultivars were sourced from across several suppliers—some cultivars are therefore likely to differ substantially in the environmental conditions experienced during cultivation and post‐harvest, including those related to agronomic and storage practices. While some endophytes are likely inherited from seed to seed (Johnston‐Monje & Raizada, 2011; Kim et al., 2022), external factors are expected to affect seed microbiome composition as the majority of the seed microbiome seems to originate from environment, especially the soil (Rodríguez et al., 2020). Soil‐derived bacteria can enter from the rhizosphere and spread throughout the plant endosphere, eventually colonizing the developing seed (Compant et al., 2011). Bacteria harboured by pollen, flowers, or fruits may also be able to colonize seed (Frank et al., 2017; Shade et al., 2017). Seed microbiome assembly has been shown to be influenced by geographic location, year‐to‐year variation in environmental conditions, and storage conditions (Chandel et al., 2021; Dutta et al., 2022; Klaedtke et al., 2016; Morales Moreira et al., 2021; Rochefort et al., 2019). For example, Markant 3, as the only cultivar in this study to be sourced from a Dutch supplier, it is not possible to attribute its unusual *Brevibacillus*‐dominated microbiome to either genotypic or environmental effects, as one confounds the other.

On the other hand, cultivars sourced from the same supplier often displayed marked differences. This may indicate a genetic effect on community assembly, which has also been reported for the seed microbiome of other crops (Adam et al., 2018; Chen et al., 2022; Johnston‐Monje & Raizada, 2011; Kim et al., 2020; Rybakova et al., 2017; Wassermann et al., 2022) and the belowground microbiome of cannabis (Comeau et al., 2020). However, environmental effects may still explain the differences observed in the study at hand, as cultivars sourced from the same supplier may still have differed in their year and location of cultivation. An experimental study that controls for environmental variation is required to reveal the influences of host genetic factors or vertical inheritance on hemp seed microbiome assembly.

Regardless of the source of variation, this study has revealed that hemp seed lots can harbour distinct seed‐endophytic bacterial communities. The observed differences in seed microbiome composition may have consequences for plant performance. Some taxa detected within the hemp seed include seed‐transmitted strains demonstrated to have plant beneficial effects, such as *Bacillus* (Mukherjee et al., 2020), *Kosakonia* (Jeong et al., 2021), *Pantoea* (Rahman et al., 2018), and *Pseudomonas* (White et al., 2018). *Bacillus* and *Pseudomonas* species have also been found to promote growth and reduce disease severity in hemp (Balthazar et al., 2021; Comeau et al., 2021). Most of the hemp seed‐endophytic microbiome remains unexplored in terms of functional characterization. Gabriele et al. (2022) found that some endophytes cultured from hemp seed were capable of producing indole‐3‐acetic acid, which can promote plant growth (Duca et al., 2014), though germination tests conducted with one such strain yielded no evidence of biostimulation (Gabriele et al., 2022).

This dataset represents a key starting point for further work on the hemp seed microbiome. A next step should be the profiling of its fungal fraction, which has received little attention beyond the isolation of taxa in culture (Scott et al., 2018). Seed‐endophytic fungi can contribute to host performance (Rétif et al., 2023) and may influence the composition of the bacterial fraction (Tannenbaum et al., 2020). Future studies should also aim to supplement taxonomic profiles with functional characterization of these seed‐endophytic communities. Culturable strains could be evaluated for plant‐growth‐promoting characteristics using in vitro tests and inoculation experiments, while community‐level function could be assessed using culture‐independent approaches, such as qPCR to quantify the abundance of bacterial genes of known functional importance to the plant (Wassermann et al., 2023), or the experimental transplant of entire microbiomes (Tosi et al., 2020). Elucidating whether members of the hemp seed microbiome contribute to plant growth and health, either at the level of individual strains or community‐level interactions, will determine the potential viability and efficacy of microbiome engineering in hemp seed. Also vital to this would be an understanding of the dynamics of the hemp microbiome beyond germination—host‐beneficial seed‐endophytes that persist within the maturing plant would represent promising candidates for further study and utilization.

## AUTHOR CONTRIBUTIONS

**Jack Davies:** Investigation; writing—original draft; formal analysis; methodology. **Sarah Hawkins:** Investigation; methodology. **Ana Winters:** Supervision; resources; writing—review and editing. **Kerrie Farrar:** Supervision; writing—review and editing; conceptualization.

## FUNDING INFORMATION

JD's PhD scholarship is awarded by the UKRI BBSRC FoodBioSystems Doctoral Training Partnership (grant number BB/T008776/1) funded by the Biotechnology and Biological Sciences Research Council, UK.

## CONFLICT OF INTEREST STATEMENT

The authors have no conflicts of interest to disclose.

## Supporting information


**Data S1.** Supporting information.

**Table S2.** Full list of amplicon sequence variants (ASVs) included within the dataset, including information on taxonomic classification, whether the ASV was classified as a contaminant and whether it was present in any of the negative control samples.

## Data Availability

Raw data of 16S rRNA gene amplicon sequences supporting the findings of this study are available in the Sequence Read Archive of NCBI under BioProject accession PRJNA1008886. The script for the analysis can be found at https://github.com/JackAlunDavies/Hemp_seed_microbiome_paper.
