# Supplementary material for: Bacterial endophytic community composition varies by hemp cultivar in commercially sourced seed
Source: Environ Microbiol Rep. 2024 Apr 22;16(2):e13259. doi: 10.1111/1758-2229.13259 (PMC11035101; doi:10.1111/1758-2229.13259)
Supplement: Supplementary file 1 — Data S1. Supporting information. [file EMI4-16-e13259-s001.docx]

**Supplementary material**

Protocol for checking sterility of final washes

2µl of the water sample was mixed with:

- Quick-Load® Taq Master Mix at X1 final concentration (New England Biolabs®)
- Primers 27F (5’-3’ AGRGTTTGATCMTGGCTCA) and 1492V2R (5’-3’ TACGGYTACCTTGTTACGACTT) each at 0.5µM (IDT, USA)

PCR reactions were performed in a 2720 Thermal Cycler (Applied Biosystems, USA):

- An initial denaturation step of 94 °C for 4 mins
- 40 cycles of denaturing at 95 °C for 30 s, annealing at 60 °C for 30 s, extension at 72 °C for 1 min
- A final extension step of 72 °C for 10 min

PCR products were mixed with BBS loading dye (MP Biomedicals™, USA) before performing agarose (1.5%) gel electrophoresis. Surface sterilisation was considered successful where bands were absent under ultraviolet light. Positive controls were included to verify the PCR.

Detailed protocol for amplicon PCR:

PCR was performed using the following components per sample:

- 2.5 µl DNA sample
- 5 µl forward primer (5' TCGTCGGCAGCGTCAGATGTGTATAAGAGACAGCCTACGGGNGGCWGCAG) for a final concentration of 0.2 µM
- 5 µl reverse primer (5' GTCTCGTGGGCTCGGAGATGTGTATAAGAGACAGGACTACHVGGGTATCTAATCC) for a final concentration of 0.2 µM
- Phusion® High-Fidelity PCR Master Mix (New England Biolabs Ltd) at a final 1x concentration

PCR reactions were performed in a 2720 Thermal Cycler (Applied Biosystems, USA):

- An initial denaturation step of 95 °C for 3 mins
- 25 cycles of denaturing at 95 °C for 30 s, annealing at 55 °C for 30 s, extension at 72 °C for 30 s
- A final extension step of 72 °C for 5 min

PCR and PCR clean up were performed using the standard protocol (16S Metagenomic Sequencing Library Preparation: Preparing 16S Ribosomal RNA Gene Amplicons for the Illumina MiSeq System, Illumina Inc), the only modification being the use of Phusion® High-Fidelity PCR Master Mix (New England Biolabs Ltd) as the polymerase mix for PCR.


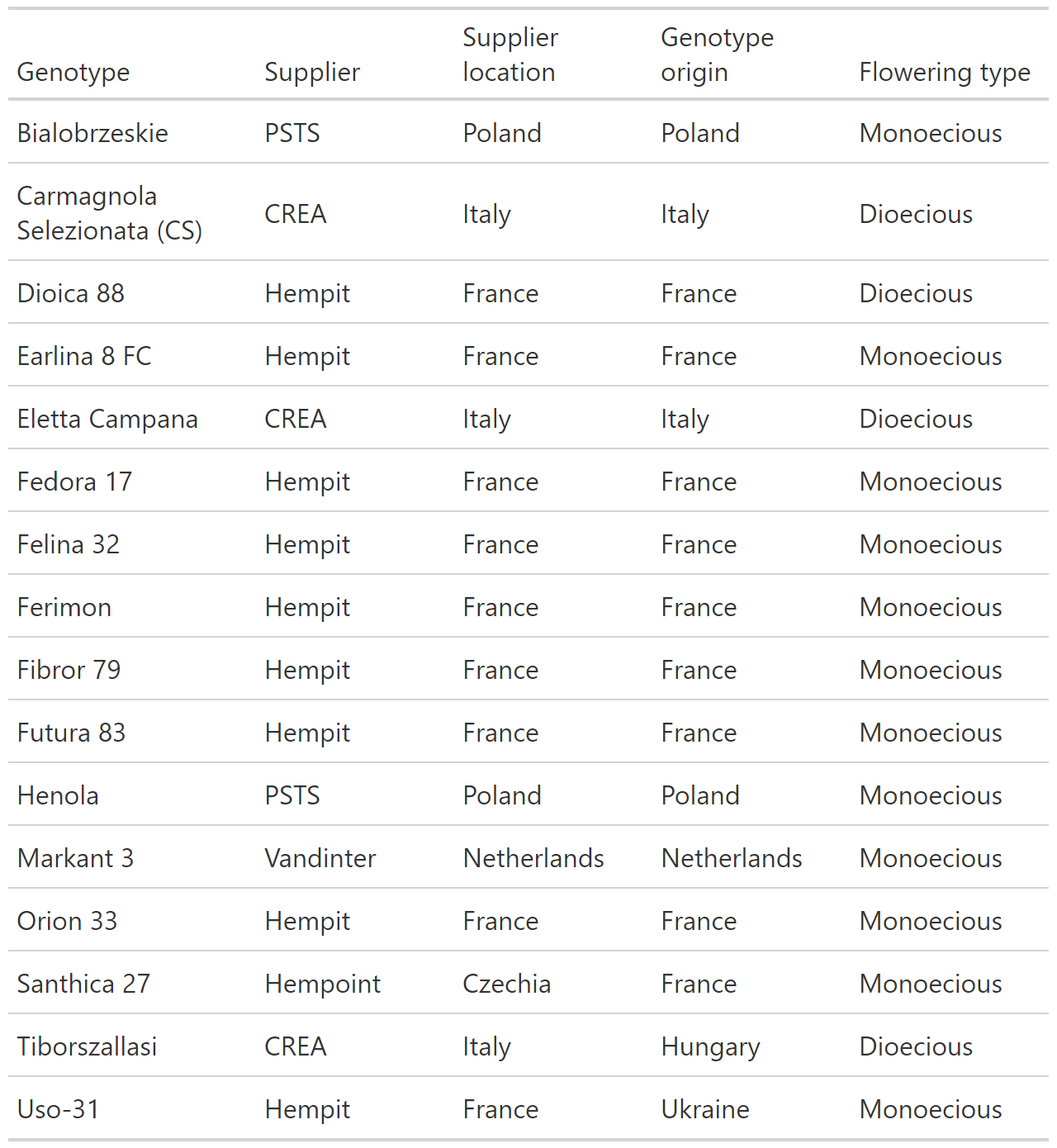


Table S1. Details of the seed material used in this study. Genotype origin and flowering type were obtained from literature searches.


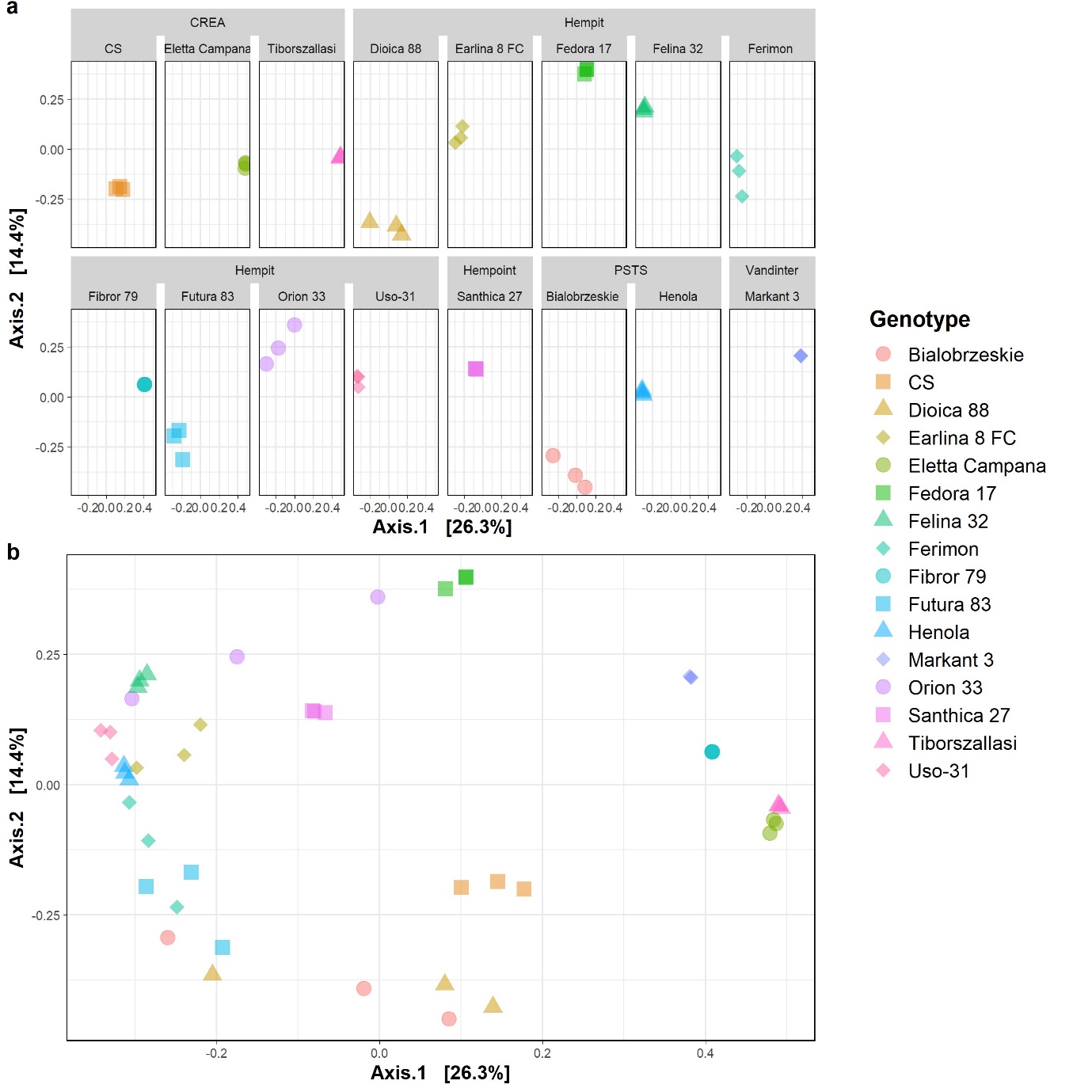


Figure S1. PCoA conducted on Bray-Curtis distances at the ASV level, presented in a faceted (**a**) and unfaceted format (**b**).


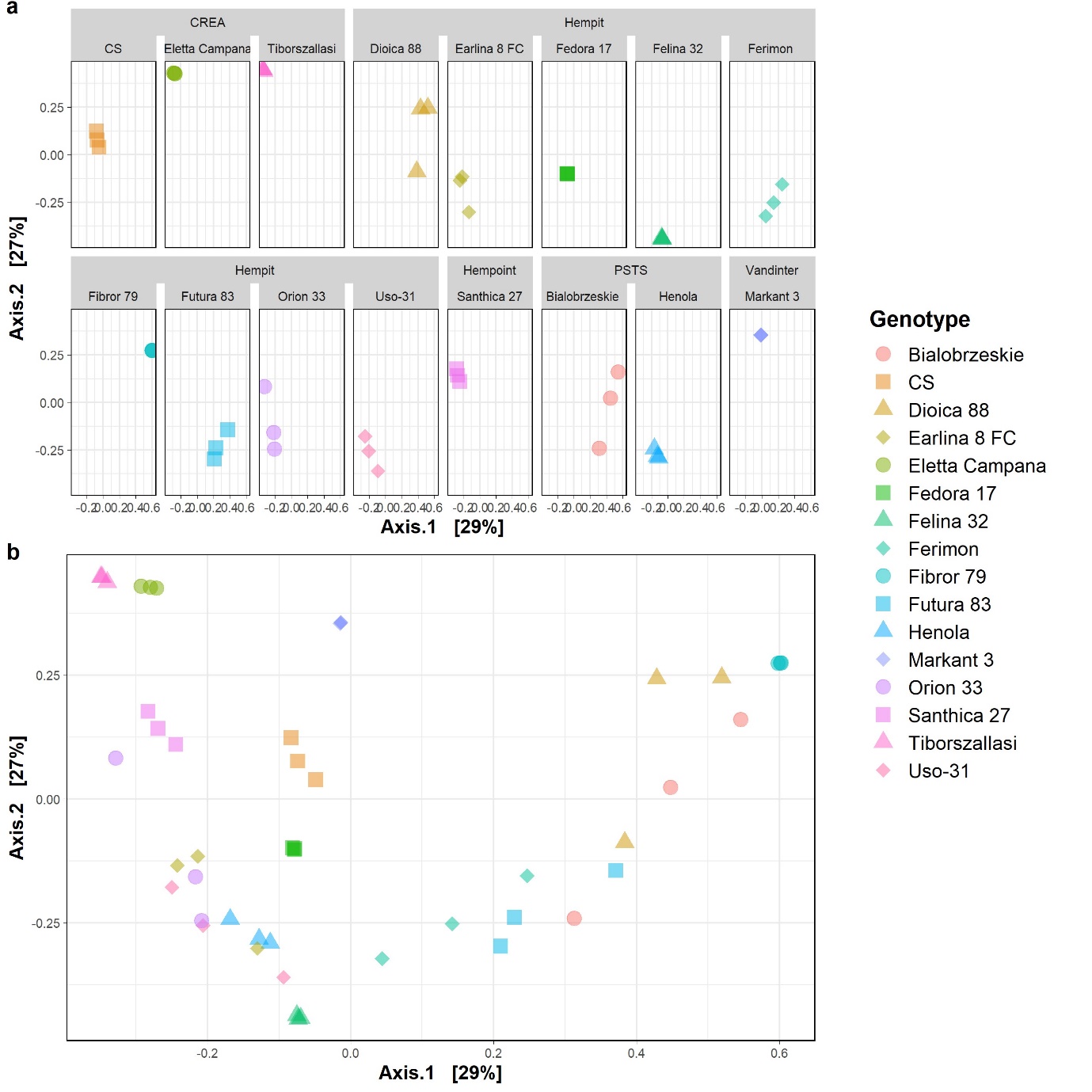


Figure S2. PCoA conducted on Bray-Curtis distances at the genus level, presented in a faceted (**a**) and unfaceted format (**b**).


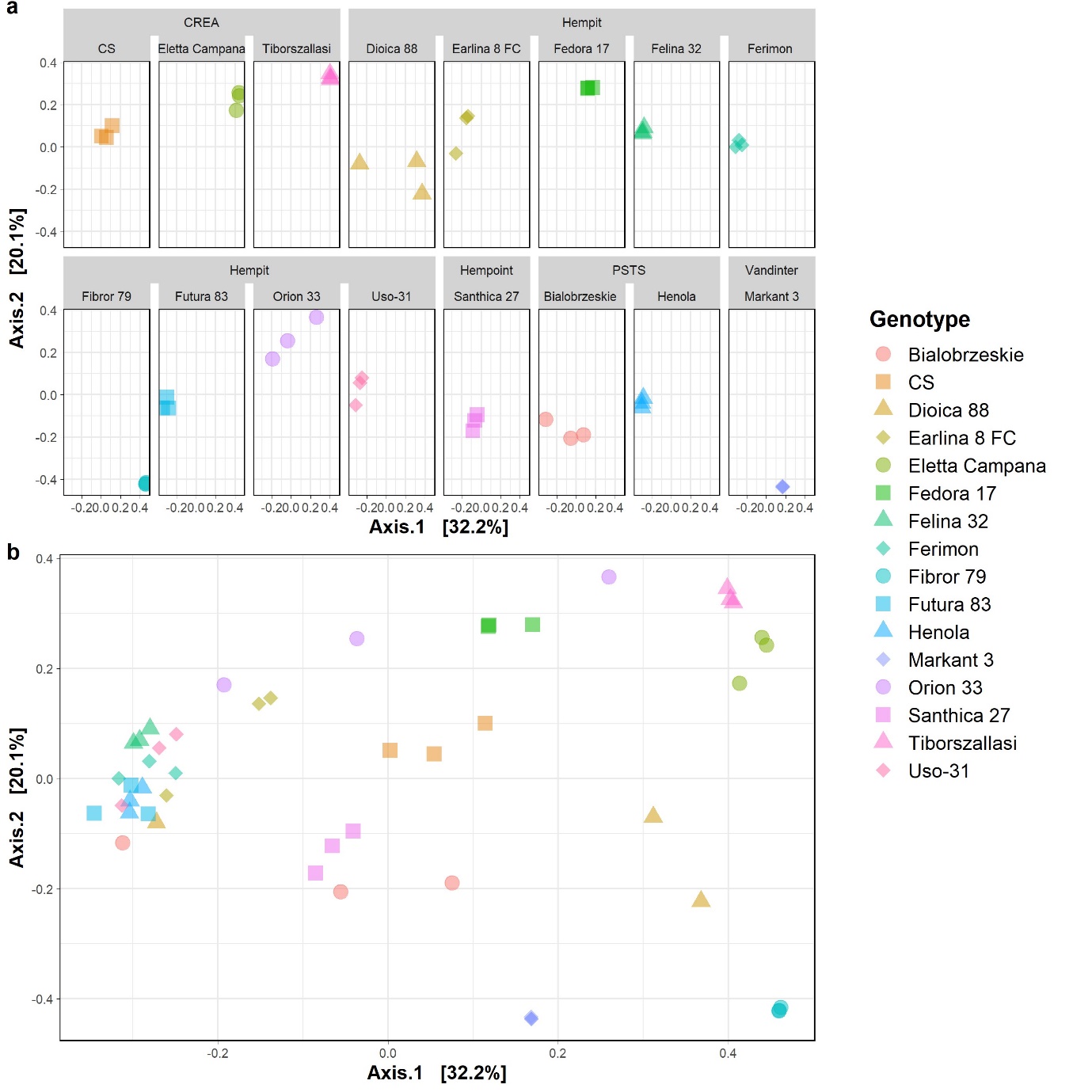


Figure S3. PCoA conducted on weighted-UniFrac distances, presented in a faceted (**a**) and unfaceted format (**b**).


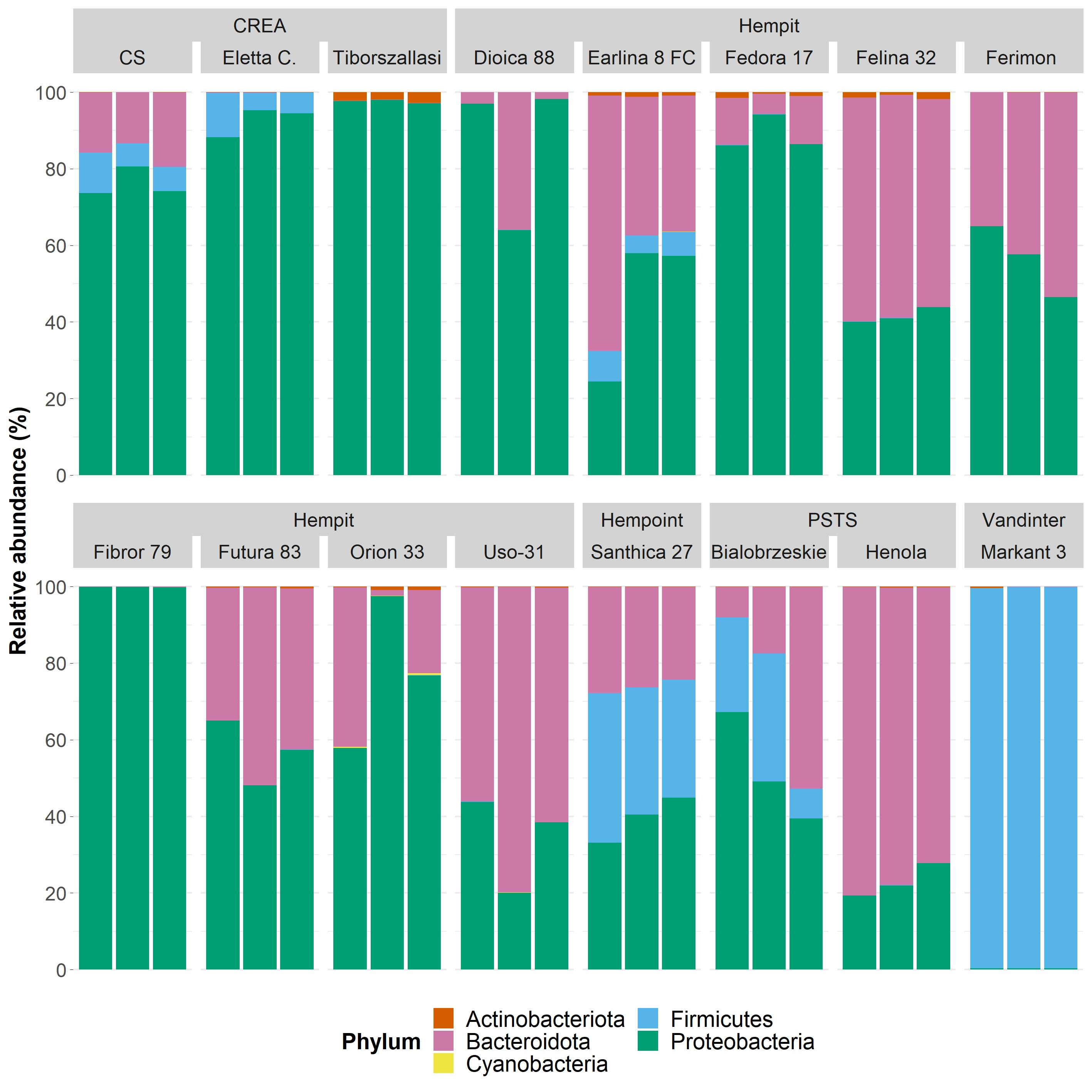


Figure S4. Relative abundance of bacterial phyla within each sample, grouped by seed supplier and hemp cultivar.


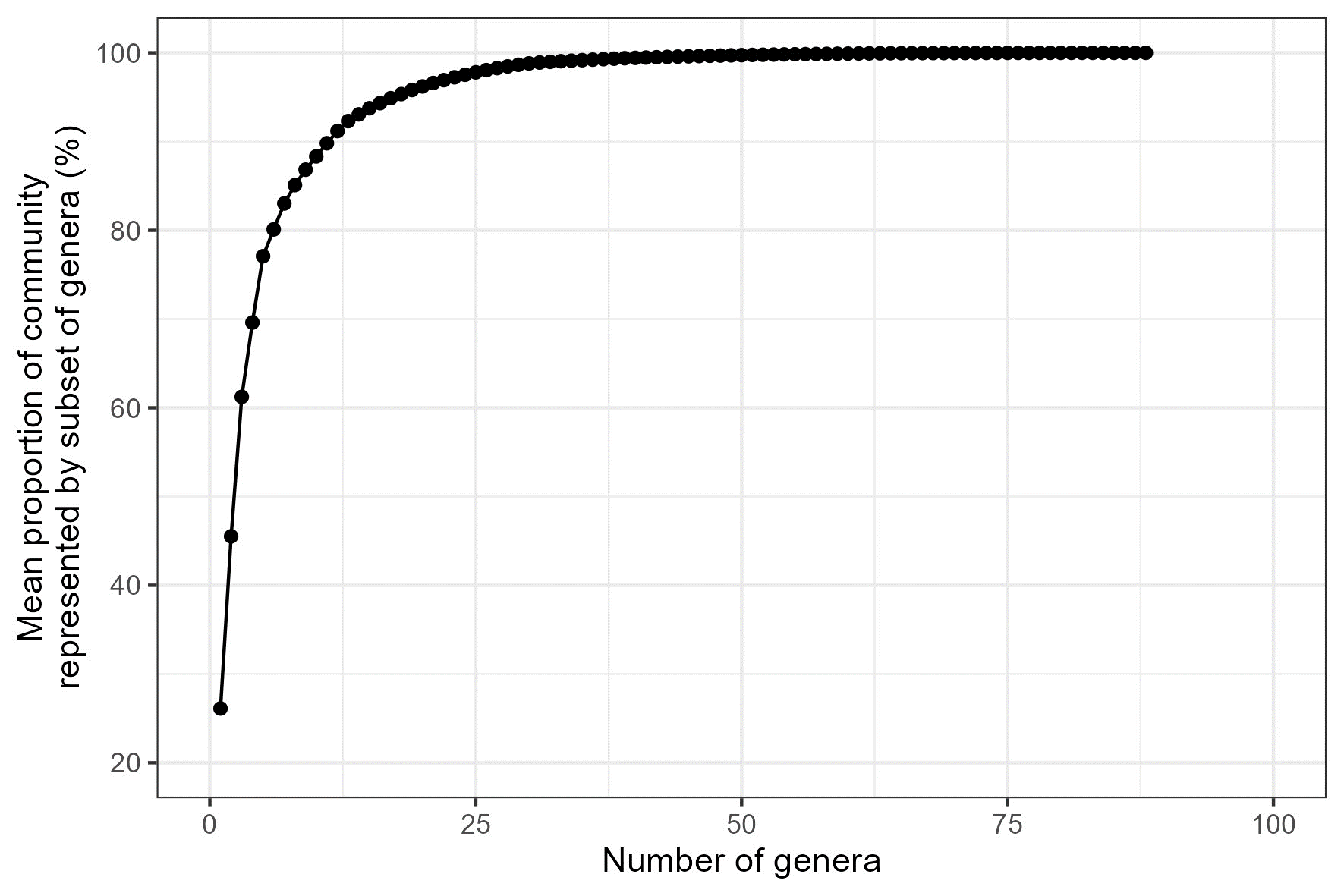
Figure S5. Mean proportion of reads per sample cumulatively represented by subsets of genera of increasing size. Genera are added to the subset in descending order of mean relative abundance.
